# Dental Splints and Sport Performance: A Review of the Current Literature

**DOI:** 10.3390/dj13040170

**Published:** 2025-04-18

**Authors:** Cornelia Popovici, Ioana Roxana Bordea, Alessio Danilo Inchingolo, Francesco Inchingolo, Angelo Michele Inchingolo, Gianna Dipalma, Ana Lucia Muntean

**Affiliations:** 1 Department of Medical Education, Physical Education Discipline, Faculty of Medicine, “Iuliu Hatieganu” University of Medicine and Pharmacy, 400012 Cluj-Napoca, Romania; popovicicornelia@yahoo.com (C.P.); anastanciu15@yahoo.com (A.L.M.); 2 Department of Oral Rehabilitation, Faculty of Dentistry, University of Medicine and Pharmacy, 400012 Cluj-Napoca, Romania; 3 Department of Interdisciplinary Medicine, University of Bari “Aldo Moro”, 70124 Bari, Italy; a.inchingolo1@studenti.uniba.it (A.D.I.); a.inchingolo3@studenti.uniba.it (A.M.I.); giannadipalma@tiscali.it (G.D.)

**Keywords:** occlusal splints, sports performance, mouth guards, dentistry

## Abstract

**Background/Objectives**: Lately, there has been a greater focus on the function of the dento-mandibular apparatus, specifically on the location of the jaw and occlusion. Given the new potential insights, the current study aimed to comprehensively analyze the published literature on the use of occlusal splints and their effects on exercise performance. **Methods**: A search was conducted on PubMed, Scopus, and Web of Science for papers published between 2014 and 2024. Starting from the 128 identified records, 28 were finally included for review. **Results**: The extensive literature review revealed significant diversity in the experimental conditions, suggesting that the occlusal splints may enhance exercise performance and support dental health. **Conclusions**: The present study highlights the growing interest in occlusal splints research and its impact on sport and exercise science. Mouthguards or occlusal splints should continue to be worn in sports with a considerable risk of orofacial injury. Regardless of how they affect performance, mouthguards or occlusal splints are crucial for athletes in many sports to prevent oral and dental injuries.

## 1. Introduction

In recent years, there has been a greater focus on the function of the dento-mandibular apparatus, specifically on the location of the jaw and occlusion. In light of the new potential insights, the current study carefully examines the published literature on using occlusal splints (OSs) and how they impact exercise performance [1].

Athletes commonly use mouthguards to prevent oral and dental injuries during training and competition. These injuries are common in contact sports and non-contact activities, including workouts. Although the data are unclear, their use has been advertised to minimize concussion incidence and severity. Mouthguards absorb impact loads, reducing the force on the teeth, bones, cranium, and soft tissue. The benefits outweigh the hazards, justifying the usage of mouthguards throughout training and competition. It is widely acknowledged that protecting against orofacial injuries has numerous benefits [2].

A strong physical condition and coordination are beneficial when participating in any activity, whether sports, business, or leisure [3,4,5]. That also involves good posture. The body’s position that preserves equilibrium under static conditions, including the spatial relationships between its anatomical components, is referred to as human posture [6]. To adapt to ongoing oscillations in the upright position, posture must be continuously adjusted [7]. The central nervous system (CNS) integrates sensory inputs, like visual, vestibular, and proprioceptive signals, from various sensors to control muscle activation [7]. According to earlier research, postural control is influenced by the state of dental occlusion [8,9].

An altered dental occlusion can impact balance stability and mobility [6]. Balance stability can be assessed, among other things, by the equal percentage distribution of body weight on the right and left forefoot and rearfoot and by the movement of the center of pressure (COP) over time; here, shorter distances correspond to more minor fluctuations [10]. Other studies support that an OC influences posture, muscular strength, and performance [11].

Other authors define occlusal splints as follows. The OC is commonly used in dental practice to manage temporomandibular disorders (TMDs), bruxism, and specific occlusal issues. They help stabilize the jaw, reduce muscle strain, and prevent excessive wear on teeth by adjusting the occlusal surface. Some types, like the Michigan splint, improve muscle relaxation and mandibular posture [12].

Occlusal splints are removable dental appliances that protect the teeth by redistributing forces and can treat conditions such as jaw pain, headaches, and joint strain due to TMD or bruxism. They maintain specific mandibular positions and reduce the load on the temporomandibular joint (TMJ) through various designs, like anterior bite planes and posterior bite planes [13]. Dental medicine is crucial for athletes’ health and well-being because up to 18% of sports-related injuries happen in the maxillofacial region. It has been recommended that sports teams should consider hiring a dentist [14].

By changing the jaw relation via a custom-made splint, the effect may be twofold: an improvement in ventilation can be achieved, and the prevailing dysfunctions in the TMJ can be regulated [15,16,17]. An advantage of using an OS is that it helps alleviate muscle pain associated with TMJ disorders by promoting muscle relaxation. A disadvantage, however, is that certain types, like soft splints, can worsen bruxism in some patients due to imbalanced posterior tooth contact [18,19].

Occlusal splints, known as night guards or bite guards, are available in several types based on the design, material, and intended function. We will analyze two main types of occlusal splints in our research, and they serve distinct yet interrelated purposes. The first type is therapeutic and is used in dentistry to manage TMD, bruxism, and other occlusal issues.

The primary types include [13,20] the following:

1. Stabilization Splints (Flat Plane Splints): Designed to cover the upper or lower arch, these splints evenly distribute bite forces to reduce muscle activity and protect the teeth. They are commonly used to manage TMD and bruxism.
2. Anterior Bite Plane Splints: These cover only the front teeth, disengaging the back teeth to prevent clenching. Due to the possibility of posterior teeth shifting with prolonged use, they are usually advised for short-term use only.
3. Repositioning Splints: These alter the jaw’s position to reduce TMJ strain and are often used to treat joint-related issues. However, they can cause permanent bite changes if used for extended periods.
4. Soft and Hard Splints: Soft splints are generally more comfortable and suitable for mild bruxism, while rigid splints are more durable and adequate for severe cases. Hybrid splints, with a hard outer layer and soft inner layer, provide a balance of comfort and durability.

These types differ in material and customization options. For example, some are fabricated from acrylic or CAD/CAM technology for precise fitting [20].

The second type, commonly known as mouthguards, is designed for athletes to protect their teeth from impact-related injuries during sports. Athletic mouthguards are categorized into three types: [21,22].

1. Stock Mouthguards: These are pre-shaped and available in multiple sizes, making them the most convenient and cost-effective choice. However, their generic fit often results in less comfort and reduced protection. Since they cannot be customized, wearers may need to clench their jaws to keep them securely in position.
2. Boil-and-Bite Mouthguards: Made from thermoplastic materials, such as ethylene-vinyl acetate (EVA), these mouthguards soften when exposed to hot water. Once heated, the user bites into the material, shaping it to match the structure of their teeth and gums. This molding process provides a more customized fit than stock mouthguards, improving comfort and protection. However, their effectiveness relies on proper shaping during the fitting process.
3. Custom-Fitted Mouthguards: These mouthguards are custom-made by dental professionals using precise impressions of the wearer’s teeth. They offer the best possible fit, comfort, and protection, but they also come at a higher cost. Due to their exceptional performance, they are especially recommended for athletes participating in high-contact sports.

While both devices serve protective functions, occlusal splints are primarily used for medical treatment, whereas mouthguards provide physical protection in high-risk activities.

Physical effort refers to the physiological and psychological energy to perform a given task, particularly in physical activities or exercises. It involves a combination of muscle contraction, cardiovascular activity, and neural coordination, requiring a measurable amount of metabolic energy [23]. Athletic performance refers to the ability of an individual to perform physical activities, particularly in sports, including strength, speed, agility, balance, and coordination. High-intensity functional training greatly impacts the development of athletes’ speed and balance [24].

Scientific research on physical effort has shown that it is closely linked to the body’s capacity to sustain and recover from activity, directly influencing performance [25]. In this context, performance is the ability to achieve a desired outcome in a physical task, often measured through speed, strength, endurance, and precision. The link between effort and performance is modulated by training intensity, individual fitness levels, and environmental factors (e.g., temperature and altitude) [26].

Understanding physical effort and performance requires an interdisciplinary approach, integrating physiology, biomechanics, psychology, nutrition, and, in this case, the dento-mandibular apparatus, to grasp how these elements combine to impact both daily activities and elite sports outcomes. The results indicated that dental occlusion differentially contributed to the dynamic stability, with an improvement when the dental occlusion was set in the correct position, disregarding the dominant and non-dominant lower limb [27]. Also, other authors agree with the fact that for both men and women, the wearing of a splint that keeps the jaw close to the centric relation improves their balance stability and increases the ROM (range of motion) of the cervical spine [1,10,28,29]. Women may have marginally different basic balance stability strategies than men regarding bipedal and unipedal standing. There were barely any differences between the two sexes in adaptation when wearing a splint. Thus, changing the jaw relation between men and women can favor and support movement potentials [10].

Sports dentistry is a developing field with an immense potential to decrease sports injuries and boost efficiency [14]. Dentists should actively assess athletes’ health status [30] because there is a correlation between the stomatognathic and musculoskeletal systems [29]. Dental health may significantly influence sports performance; thus, proper preventive measures could enable athletes to sustain their training and competition schedules without disruptions caused by dental discomforts [30]. Consequently, enhancing dental state evaluations is strongly advised in the medical monitoring of athletes [31].

Prior research indicates that the oral occlusion status affects postural control [6,25,26]. Dental occlusion and the relative position of each tooth also determine the mandible posture [27]. However, oral health is also an essential component of overall health in athletes. Poor oral health can induce systemic inflammatory responses, affect athletes’ physical fitness, and even negatively influence their athletic performance and balance [32].

Because tooth occlusion may impact muscle strength somewhere else in the body, splints have also been used for better athletic results [14,33,34]. In healthy patients, occlusal splints worn in a centric relation position have an ergogenic effect by enhancing shoulder strength and muscular activation. Splints are vital for sports where upper-body strength is essential for performance (like boxing) [14]. However, the athlete and the potential intervention to improve performance in high-level sports should always be regarded individually [35].

Dancers’ general efficiency and neuro-muscular synergy were enhanced using a customized dental apparatus for six months, during which electromyography (EMG) data and balancing tests were obtained. The orthotic could improve postural balance and classical ballet performance when applied to participants exhibiting asymmetrical muscle activation [26]. The EMG was successfully used to diagnose TMD in basketball athletes. According to the results, EMG has a high degree of discriminative power, especially regarding cases of muscular TMD. Asymmetry metrics are vital for diagnosis when paired with clinical examination, and key indicators like overall scores, such as: percent overlap coefficient between masseter muscles and anterior temporalis (POC-TA), torsional attitude of the mandibula in horizontal position (TORS), asymmetry (ASIM), and anterior temporalis activity offer insightful information. The diagnostic accuracy is improved using latent EMG variables, especially when identifying borderline cases. Furthermore, healthy athletes had more consistent and higher SCORE values, making it easier to identify using EMG [36].

The fascial system is also essential because it comprises mechanical receptors and has an autonomous contractile ability that affects the tension of the fasciae; in addition to its ability to passively distribute stress in the body muscles when mechanically triggered [29]. The posture of the body appears to be impacted by these tensions [29]. At the same time, research about the influence of occlusion splints on the rate of force development (RFD) and maximal strength tests was confirmed. Individualized splints increase performance in jumping and strength tests [37].

Research on physical performance analysis and the dento-mandibular apparatus has made significant progress, as proven by recent publications. This study aims to thoroughly assess the literature on occlusal splints and athletic performance, considering new possible insights.

## 2. Materials and Methods

### 2.1. Protocol and Registration

This systematic review was conducted according to the Preferred Reporting Items for Systematic Reviews and Meta-Analyses (PRISMA). The PICO question was used to determine the eligibility requirements, which addressed the following guidelines: does dental occlusion affect athletes’ performance?

P—patients—athletes; 

I—dental occlusion interferes with athletes’ performance; 

C—normal occlusion and dental anomalies; 

O—evaluation. 

The main goal was to explore how dental occlusion relates to sports performance and if splints can benefit athletes.

### 2.2. Search Processing

A search was conducted on PubMed, Scopus, and Web of Science for papers published between 1 January 2014 and 1 May 2024 to identify research that assesses the usage of occlusal splints in physical activity and its implications on sports performance. Boolean keywords were used in the search strategy: (“dental occlusion”) AND (“sport performance”) AND (“occlusal splints”) AND (“sports dentistry”). We chose key terms that closely coincided with our study’s purpose, which aimed to investigate the role of dental occlusion in sports performance. (Table 1).

### 2.3. Eligibility Criteria and Study Selection

The selection process consisted of two stages: evaluating the abstract, title, and the entire material. Inclusion criteria included open-access studies on dental occlusion and sports performance, reviews, English-language publications, and full-text articles. Documents that were not compatible with the required specifications were not included.

Excluded papers included research methodologies, conference presentations, in vitro or animal experiments, meta-analyses, and those lacking original data and full text. The initial search yielded relevant titles and abstracts. Complete articles from relevant research were gathered for analysis. Two reviewers (P.C. and A.M.) evaluated the retrieved studies for inclusion based on the abovementioned criteria.

### 2.4. Data Processing

Based on the selection criteria, two reviewers (P.C. and A.M.) separately accessed the databases to collect the studies and provide a quality grade. During the screening process, publications that did not correspond with the examined themes may have been excluded due to disagreements. After determining that the publications met the predetermined inclusion criteria, the entire text of each was read.

## 3. Results

### 3.1. Selection and Characteristics of Study

A total of 128 publications were found using the electronic database search (Web of Science = 36, PubMed = 65, and Scopus = 28)

**Figure 1:**
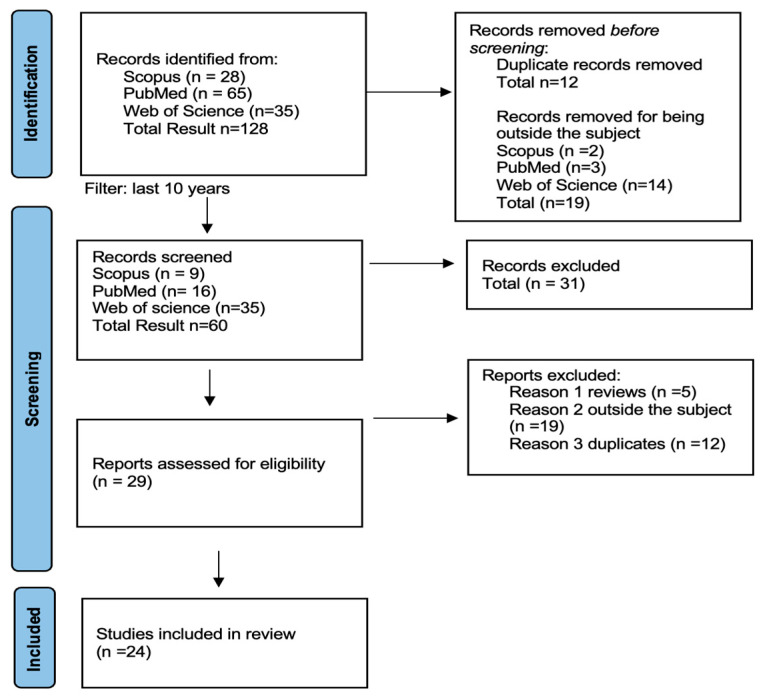
Prisma flow diagram [38].

The first search yielded 128 studies. Only articles published within the last ten years were included using the inclusion and exclusion criteria. A total of 64 remained. After an additional examination, only human studies were chosen, and after removing duplicates from the tree databases, articles were manually picked, resulting in 24 papers at the end of the research (see Figure 1).

### 3.2. Synthesis of Results

The search results are organized in the following table (see Table 2) into parts based on the many characteristics of exercise performance examined in the included research (see Table 2).

The included studies tested many aspects of physical performance, including aerobic and anaerobic capacity, maximum and explosive power, force, and strength, as well as evaluated other indicators, all related to occlusal splints.

## 4. Discussion

Dental occlusion or mouthguards are essential for athletes in numerous sports to protect against oral and dental injuries, independent of their impact on performance. Orofacial injuries occur in 39.1% of athletes who participate in contact sports, with injury types varying based on the nature of the activity, competitive level, age, group sex, and additional influencing factors [54]. For example, among professional handball players, 49% experienced head and/or facial injuries, while 22% reported dental trauma, with 76% leading to complications [55]. Another study of 169 ice hockey players in Canada found that 45.6% never wore a mouthguard, 23.1% always wore one, 14.8% occasionally wore one, and 16.5% only wore one when required. Additionally, 57.7% of players were hit by a stick, 46.2% by a puck, and 25% experienced a body check from an opponent. These findings highlight the importance of improving mouthguard designs to enhance protection, comfort, and player compliance [55]. Goud et al. advocate using occlusal splints to reduce the risk of sport-associated dental trauma [56].

Mouthguards are essential for protecting dentition and mouth face tissues, but their application has been constrained by various drawbacks and restrictions [53]. Utilizing a mouthguard to adjust jaw positioning for enhanced performance is not new. A previous study [57] showed positive effects on the isometric strength in the neck and head. Since medicine evolved, neuromuscular dentistry techniques have become more advanced. A survey of male college athletes performing sports like basketball, wrestling, mixed martial arts, and lacrosse emphasized that using a mouthguard can influence athletic performance in the peak power and consistent power output [58].

An improvement while using the occlusal splint was observed in professional basket players, mainly the force of the quadriceps muscle [59]. Using a splint to keep the mandible near the centric relation improves the balance, stability, and range of motion (ROM) in the cervical spine for both male and female participants. Women could experience slightly alternative balance firmness methods than male participants for two-legged and one-leg standing. Both sexes adjust similarly to using a brace. Altering the jaw alignment in healthy individuals may help release movement potentials, simplifying everyday activities and sports motions. Göttfert et al. [10] found in their study that for both men and women, wearing a splint that retains the mandibula, bringing the jaw closer to the centric relation, enhances balance stability and increases the cervical range of motion

Women may employ slightly varied fundamental strategies for balance and stability compared to men during bipedal and unipedal standing. However, the differences in splint adaptation between genders were minimal. Thus, modifying the jaw relationship in men and women may favor and support movement potentials. An improvement in the body posture of pilots was observed [60]. Minor adjustments in the positioning of the lower jaw can influence muscle activation, resulting in a better-balanced force production [61,62].

Prior studies [41,63,64] have proven the effect of occlusion factors on muscular chains. Improved balance, upright posture, and symmetrical walking patterns have been observed. These movements typically require minimal muscular activity. In maximal strength testing, muscles are engaged to produce maximum force and power. Minor adjustments in the positioning of the lower jaw can influence muscle activation, resulting in better-balanced force production [41,63,64]. The use of customized splints was evaluated on triathletes over four months, and enhancements in balance and biting function were found, though were not necessarily aligned with improvements in sports performance [65].

Dias et al., examining the impact of dental occlusion on shoulder strength, indicated that an OS provides a beneficial ergogenic effect on shoulder and arm strength in healthy individuals compared to a condition without a splint. These findings could have implications for sports and physical activities that require significant upper-body strength, particularly in the arms and shoulders [14]. However, Baum et al. [66] determined that the use of a dental splint does not significantly impact the rehabilitation of the glenohumeral internal rotation deficit in female volleyball players.

If an athlete has a malocclusion, they should undergo clinical and instrumental examinations to determine the benefits of using an oral splint for their discipline [67].

The most common reasons for not wearing a mouthguard are discomfort and challenges with breathing, speaking, and swallowing during physical activities [68,69]. Athletes in another study also experienced pain and problems breathing, which were caused by the continual masticatory force necessary to keep mouthguards in position due to insufficient retention [70]. In the study performed by Biagi R. et al. [71] the primary reasons for not wearing a mouthguard among athletes were lack of interest (12%) and insufficient information (9%). Additionally, 2% believed a mouthguard could not be used during ongoing orthodontic treatment, while 1% avoided it for esthetic reasons. Furthermore, 5% stated that they refrained from using a mouthguard because it negatively impacted their sports performance, and 3% reported experiencing breathing difficulties. However, in kickboxing, the use of a mouthguard is mandatory.

Vertical jump testing and the countermovement jump (VSJ and CMVJ) are commonly utilized to evaluate explosive power in the lower limbs [72]. Some studies have found enhancements in jump performance after the implementation of OSs for the VJ and CMVJ [45,70,73], while others observed improvement in lower body power for men, [74], or no significant differences [75].

Militi A et al. concluded that the application of an occlusal splint should be assessed across various sports, as athlete’s occlusion, temporomandibular joint conditions, and physical exertion levels vary [67].

Oral health plays a crucial role in physical and sports performance, and occlusal splints can enhance athletic performance. Cesanelli et al. emphasized that oral splints should be used to improve oral health in athletes and as a possible tool to enhance sports performance. They also recommend teamwork between medical staff, athletes, and coaches to establish each athlete’s peculiarities, focus on sports performance only, and consider the occlusal splints. Greater attention should be directed to athletes’ oral health to enhance their overall well-being and quality of life and potentially improve their sports performance [1].

Additionally, coaches, sports clubs, and federations need to recognize the significance of implementing oral health prevention programs for athletes [28], as well as the impact of occlusion on posture and the relationship between occlusal variations and changes in balance [28,29,30]. The mandible position influences physical performance [29], even if the OS is custom-made or commercial [1].

## 5. Conclusions

This study underscores the critical role of occlusal splints in maintaining and improving oral health by addressing issues such as bite misalignment, bruxism, and TMD. Occlusal splints also act as a preventive measure against dental wear and potential long-term complications. Regular dental check-ups and assessments are essential to determine the appropriateness and effectiveness of occlusal splints for individual patients, ensuring personalized treatment plans that optimize their therapeutic benefits.

In contrast, mouthguards are primarily designed to protect athletes’ teeth and oral structures from impact-related injuries. Their mandatory implementation in high-risk sports remains crucial to injury prevention strategies. While distinct in their primary function—occlusal splints focusing on therapeutic intervention and mouthguards on injury prevention—both devices contribute to overall oral stability and influence jaw positioning.

The findings from this study emphasize the need for standardized research methodologies to assess the full impact of occlusal splints on oral health, neuromuscular function, and sports performance. Future investigations should optimize design features, improve material properties, and refine clinical recommendations to enhance their effectiveness for therapeutic and protective purposes.

## Figures and Tables

**Table 1:** Database search indicators.

Database Search Indicators	
Article screening strategy	KEYWORDS: A (“dental occlusion”) AND B (“sport performance”) AND C (“occlusal splints”) AND (“sports dentistry”).
	Boolean Indicators: A AND B AND C AND D.
	Timespan: 2014–2024
	Electronic Databases: PubMed; Scopus; and WOS.

**Table 2:** Data analysis of included studies.

Year	Authors	Category	Intervention	Results	Oral Splint Category, Occlusal Modifications
2014	Allen et al. [39];	21 recreationally male-trained colleges.	Maximum countermovement vertical jump; one repetition maximum bench press.	Slight increases in vertical jump (PF = 0.08) and bench press (RFD *p* = 0.45) during the countermovement (CMVJ *p* = 0.13) were observed in the mouthpiece condition, and these improvements were minimal and not statistically significant.	Mouthpiece.
2015	Golem and Arent. [40];	20 male collegiate athletes.	Muscular power, dynamic balance, flexibility, agility, and muscular strength.	The jaw-repositioning technique did not affect performance. Muscular power *p* = 0.78, dynamic balance *p* = 0.99, agility *p* = 0.22, and muscular strength *p* = 0.47.	Placebo mouth guard, a self-adapted jaw-repositioning mouth guard, and a custom-fitted jaw-repositioningmouth guard.
2015	Ringhof et al. [35];	14 male professional golfers.	Shot precision and shot length over three different Distances.	Statistical analysis showed that oral motor activity did not affect golf shot precision or length for 60 m, 160 m, or driving distance. Only Pin length at 160 m was statistically significantly impacted by oral motor tasks (*p* = 0.043, η2p = 0.22).	Oral splint.
2015	Maurer et al. [41];	20 healthy young recreational runners.	Kinematic 3D analysis of running; Wingate test 30 s; and Spirometry.	There was no discernible variation in running pace among the four situations (*p* = 1.00) Under splint conditions, a more symmetrical running pattern was discernible.	Occlusal splint in the lower jaw.
2016	Battaglia et al. [42];	18 physically active subjects, 18 sedentary subjects.	Cervical range of motion.	Occlusal splints failed to improve the physical performance significantly; cervical range of motion *p* ≥ 0.05.	Custom.
2016	Patti et al. [29];	60 male football players.	Squat jumps with different mandible positions: mouth closed and mouth open.	There was a statistically significant difference between the outcomes (*p* < 0.0001).	Occlusal factors.
2016	Busca et al. [43];	28 physically active male subjects.	Jump ability and isometric maximal strength tests.	Significantly higher performance for handgrip force (*F*_(2,54)_ = 32.71, *p* ≤ 0.05) and all variables of the back-row exercise (*F*_(2,54)_ = 13.74, *p* ≤ 0.05) and countermovement vertical jump (*F*_(2,54)_ = 8.04, *p* ≤ 0.05), as peak force, while wearing the mouthpiece.	Customized bite-aligning mouthpiece.
2016	Ringhof et al. [44];	12 healthy young adults.	Dynamic stability, bite forces.	Both conditions led to a considerable rise in bite forces (*p* = 0.011). However, the findings demonstrated that, according to the factors under investigation, biting with a submaximal force had no discernible effect on the recovery behavior of healthy young adults (*p* = 0.515).	Hydrostatic splints.
2017	Fisher, Weber and Beneke [45];	23 physically active subjects.	Wingate test 30 s.	Results showed moderate to perfect correlations between all splint conditions, with coefficients of variation ranging from 1.3% to 6.6%.	Custom, upper jaw.
2017	Golem and Davitt and Arent [46];	20 College-aged male athletes.	Respiratory flow dynamic tests at rest; maximal treadmill test.	This study’s OTC self-adapted jaw-repositioning mouthguards did not improve aerobic performance (*p* = 0.35).	Placebo mouthguard, self-adapted jaw-repositioning mouthguard, custom-fitted jaw-repositioning mouthguard.
2018	Dias et al. [6];	13 national-level male shooters.	Surface electromyography, shooting score.	No evident changes in EMG activity (*p* = 0.069).	Custom splints in the upper jaw.
2018	Maurer et al. [37];	23 healthy, mid-age recreational runners.	Squat jump, countermovement jump, and drop jumps from four different heights and three maximal strength tests: trunk flexion and extension, leg press in both legs.	The squat jump, countermovement leap, drop jumps, trunk extension, leg press force, and rate of force generation all showed significant results. A 3% to 12% improvement is the result of the following circumstances.	Custom splints in the lower jaw.
2018	Leroux et al. [15];	7 members of the “Pôle France Aviron”.	Body balance, paravertebral muscle contraction symmetry, and muscular power.	Negative impacts of an occlusal disturbance on the athletic performance of young elite rowers caused a notable 17.7% drop in the athletes’ muscle strength (*p* = 0.030).	Occlusal silicone splint.
2019	Dias et al. [14];	14 male healthy subjects.	Isokinetic strength was evaluated in shoulder abduction/adduction and arm external/internal rotation tests.	There were no changes in muscle activity in the middle deltoid under either condition. There was a substantial increase in the anterior deltoid (*p* < 0.01) and pectoralis major (*p* < 0.01) muscular EMG activity in the OS condition compared to other conditions. In the lower trapezius, the OS condition significantly increased the peak EMG compared to the N condition (*p* < 0.05).	Occlusal splints in the upper jaw.
2020	Julia-Sanchez et al. [27];	30 physically active subjects.	Excursion Balance Test.	The Star Excursion Balance Test composite score was considerably higher for measures taken in cotton rolls mandibular position (*p* < 0.001) and in patients with proper occlusion (*p* = 0.04).	Occlusal splints.
2021	Kinjo et al. [47];	3 male participants.	Dental occlusion tests were performed with the MG sensor.	The MG sensor is essential for detecting personal teeth-clenching habits during exercise. No significant differences were observed between either sensor (*p* > 0.005). The EMG and MG sensor outputs differed significantly (*p* < 0.05).	Wearable MG device with force sensors on both sides of the maxillary to monitor teeth clenching.
2021	Didier et al. [28];	20 ballet dancers.	Electromyography (EMG) data and balance tests.	All of the performed EMG tests and the Flamingo Balance Test revealed statistically significant adjustments (*p* < 0.001 for scans 9 and 11). There were just three dancers whose asymmetry of muscle activation remained unchanged.	Customized occlusal splint.
2021	Carbonari et al. [48];	18 athletes.	Surface electromyography kinesiography, the squat jump and countermovement jump, and the handgrip test.	Applying an occlusal splint and Taopatch (R) devices either separately or together instantly impacted the occlusal postural muscles’ strength and balance. The squat jump increased the height of 10–14 mm. The biting and Taopatch^®^ devices alone resulted in a more substantial handgrip (~5 lbs).	Customized soft occlusal splint to the lower arch, nanotechnological devices.
2022	Cardoso et al. [49];	16 middle- and long-distance runners.	7 × 800 m intermittent running.	No differences between placebo and lower jaw advancer were found (e.g., 52.1 ± 9.9 vs. 53.9 ± 10.7 mL·kg^−1^·min^−1^ of oxygen uptake, 3.30 ± 0.44 vs. 3.29 ± 0.43 m of stride length and 16 ± 3 vs. 16 ± 2 Borg scores).	Two lower intraoral splints, custom (a placebo and a lower jaw advancer.
2022	Inchingolo et al. [50];	25 athletes (20 football players).	Electromyographic recording using two clamping tests.	Splint treatment showed an overall efficacy of 72%, statistically significant efficacy on rebalancing of the barycenter.	Custom occlusal splint.
2022	Parini et al. [51];	16 track and field athletes.	Countermovement jump; drop jump; 10 m and 30 m sprint tests.	The majority of the athletes who were evaluated performed better in countermovement jump, 10 m, and 30 m sprint tests during training sessions with occlusal splints than athletes without them, although it was not able to show an increase in sports performance.	Custom occlusal splint.
2022	Dias et al. [52];	22 male amateur rugby players.	1 bench press test.	Controlled mouthguards increase peak power in the ballistic bench press exercise (*p* < 0.05).	Controlled mouthguard, jaw in centric relation, non-controlled mouthguard, occlusal splint.
2022	Kalman et al. [53];	A 3D volumetric skull with teeth was designed.	Static structural and mechanical analysis.	The hybrid occlusal splint-mouthguard minimized jaw displacement during chewing, reducing stresses in maxillary and mandibular teeth. Compared to the custom design, the hybrid design showed a greater degree of stress on its occlusal section (7.05 MPa) than the MG design (6.19 MPa).	Conventional custom mouthguard, hybrid occlusal splint.
2022	Göttfert et al. [10];	41 male and 50 female subjects.	Cervical spine range of movement, balance stability.	Bipedal and unipedal standing demonstrated a greater range of motion extension and improved balance stability in females by up to 4°. When wearing a splint, there are very few differences in adaptation between the sexes (men *p* ≤ 0.01 and *p* ≤ 0.001 and women *p* ≤ 0.001 and 0.04).	Custom splints.

## Data Availability

Not applicable.
